# Innate Lymphoid Cells and Natural Killer Cells in Bacterial Infections: Function, Dysregulation, and Therapeutic Targets

**DOI:** 10.3389/fcimb.2021.733564

**Published:** 2021-11-05

**Authors:** Noha Mousaad Elemam, Rakhee K. Ramakrishnan, Jennifer E. Hundt, Rabih Halwani, Azzam A. Maghazachi, Qutayba Hamid

**Affiliations:** ^1^ Sharjah Institute for Medical Research, College of Medicine, University of Sharjah, Sharjah, United Arab Emirates; ^2^ Department of Clinical Sciences, College of Medicine, University of Sharjah, Sharjah, United Arab Emirates; ^3^ Lübeck Institute for Experimental Dermatology, University of Lübeck, Lübeck, Germany; ^4^ Prince Abdullah Ben Khaled Celiac Disease Chair, Department of Pediatrics, Faculty of Medicine, King Saud University, Riyadh, Saudi Arabia; ^5^ Meakins-Christie Laboratories, McGill University, Montreal, QC, Canada

**Keywords:** innate lymphoid cells (ILCs), natural killer cells (NKs), bacterial infection, mucosal immunity, intracellular bacteria, extracellular bacteria

## Abstract

Infectious diseases represent one of the largest medical challenges worldwide. Bacterial infections, in particular, remain a pertinent health challenge and burden. Moreover, such infections increase over time due to the continuous use of various antibiotics without medical need, thus leading to several side effects and bacterial resistance. Our innate immune system represents our first line of defense against any foreign pathogens. This system comprises the innate lymphoid cells (ILCs), including natural killer (NK) cells that are critical players in establishing homeostasis and immunity against infections. ILCs are a group of functionally heterogenous but potent innate immune effector cells that constitute tissue-resident sentinels against intracellular and extracellular bacterial infections. Being a nascent subset of innate lymphocytes, their role in bacterial infections is not clearly understood. Furthermore, these pathogens have developed methods to evade the host immune system, and hence permit infection spread and tissue damage. In this review, we highlight the role of the different ILC populations in various bacterial infections and the possible ways of immune evasion. Additionally, potential immunotherapies to manipulate ILC responses will be briefly discussed.

## 1 Introduction

Bacterial infections were, are and will continue to remain a pertinent health challenge and burden. The human body is under constant exposure to a plethora of bacteria, including commensal and pathogenic bacterial species. The immune system is equipped to ward off pathogenic bacteria while maintaining symbiosis with the commensal flora. However, some bacterial species have evolved to evade the host protective responses and establish infections.

Innate lymphoid cells (ILCs), are innate lymphocytes that lack adaptive antigen receptors (Artis and Spits, 2015). Nevertheless, they are equipped with a wide array of activating and inhibitory receptors. During fetal development, a subset of ILCs functions as lymphoid tissue-inducer cells (LTi cells), that induce lymphoid organogenesis and are involved in the formation of secondary lymphoid organs (Mebius et al., 1997). Although ILCs do not undergo antigen priming, they immediately respond in an antigen-independent manner either upon engaging their germline-encoded receptors or through cytokine stimulation resulting in effector cytokine secretion (Glatzer et al., 2013).

ILCs are basically classified into three subgroups, namely group 1 innate lymphoid cells (ILC1s) including natural killer (NK) cells and interferon (IFN)-γ secreting ILC1s, group 2 innate lymphoid cells (ILC2s), and group 3 innate lymphoid cells (ILC3s), based on their lineage-defining transcription factors and cytokine secretion profiles (Spits et al., 2013; Elemam et al., 2017). These subgroups are largely considered as the innate counterparts of CD4^+^ T helper (Th)1, Th2, and Th17 cells respectively, while the NK cells are analogous to CD8^+^ cytotoxic T cells (Vivier et al., 2018). As such, the differentiation and function of ILC1s in response to IL-12 and IL-18 depend on the expression of transcription factor T-box expressed in T cells (T-bet, also called as TBX21), while NK cells depend on the transcription factor eomesodermin (Eomes) resulting in the production of IFN-γ and tumor necrosis factor (TNF)-α (Spits et al., 2013). Like Th1 cells, ILC1s respond to intracellular pathogens such as bacteria and viruses. On the other hand, ILC2s express GATA binding protein 3 (GATA3) and produce cytokines IL-4, IL-13, IL-5 and IL-9, in response to IL-25 and IL-33. Together with Th2 cells, ILC2s are involved in responses to extracellular parasites/helminths, allergens and tissue repair. ILC3s express retinoic acid-related orphan receptor gamma t (ROR-γt) in response to IL-23 and IL-1β, and produce IL-22, IL-17, granulocyte-macrophage colony-stimulating factor (GM-CSF), and lymphotoxin (LT)-α1β2. ILC3s and Th17 cells respond to extracellular pathogenic bacteria and fungi. Therefore, the various ILC subsets, respond to a wide array of pathogens ranging from bacteria, parasites, viruses and fungi, albeit in a subset-specific manner. It is also worth mentioning that plasticity exists among these cells in order for them to adapt their transcriptional profile to the local microenvironment cues and specific cytokine exposure (Vivier et al., 2018).

In contrast to T cells, ILCs respond quickly to stress signals from tissue-resident cells. Their production of effector cytokines helps activate and regulate the activity of both innate and adaptive immune cells such as T cells, B cells, dendritic cells (DCs), eosinophils, neutrophils, macrophages, and epithelial cells (Artis and Spits, 2015). ILCs work in synergy with T cells where they interact and cross-regulate each other, thus amplifying their response. Nevertheless, they also compete with each other for the same growth factors and inducer cytokines.

Developmentally, ILCs are programed to migrate, differentiate and populate mucosal tissues and lymphoid tissues. They are primarily tissue-resident cells that are constitutively present in mucosal tissues, such as the respiratory and gastrointestinal tracts. The presence of ILCs in close proximity to mucosal barriers leads to their exposure to a wide variety of both commensal and pathogenic bacteria. Their interaction with the microbiome is important for the maintenance of tissue homeostasis. The orchestration of the relationship between host and commensal bacteria in turn influences the homeostasis of ILCs (Sonnenberg and Artis, 2012). Further, these cells are characterized by their rapid response in mucosal defense against pathogenic bacteria and in orchestrating other immune cells.

ILCs are crucial for mucosal tissue homeostasis and any dysregulation of these cells could lead to a broad spectrum of diseases, including bacterial infections. Multiple interactions with various cell types regulate the function of ILCs. For instance, neuroimmune circuits have been shown to integrate extrinsic environmental signals such as light-dark cycles and nutrient intake, to orchestrate ILC responses at barrier surfaces to harmonize immunity. There is a reported interaction between ILCs and the enteric nervous system (ENS), leading to ILC activation and cytokine secretion (Han et al., 2019). On another note, neurons and enteric glial cells (EGCs) were found to interact with ILC3s through neurotrophic factor signals, thus protecting the intestinal lining against inflammation and microbial infection (Bessac et al., 2018). Also, enteric neurons express and sense cytokines such as TSLP, IL-4, and IL-31, where they crosstalk with ILCs, thus promoting a type 2 response (Wilson et al., 2013; Oetjen et al., 2017). In addition, the circadian rhythm controls ILC2 and ILC3 activation in the intestine to regulate intestinal homeostasis and gut defense (Godinho-Silva et al., 2019; Talbot et al., 2020). There is a proposed connection between ENS, gut microbiota and ILC (Rolig et al., 2017). Furthermore, the neuropeptide vasoactive intestinal peptide (VIP) expressed by enteric neurons exerts both stimulatory and inhibitory effects on CCR6+ ILC3s and its functional role appears to be context-dependent and impacted by the commensal microbiota. On the other hand, a direct stimulation of ILC2s by neurons through the released neuronal messenger neuromedin U was reported, causing an induced immune response (Han et al., 2019). In this review, we highlight the role of ILCs in various bacterial infections and their possible evasion by bacteria. Additionally, potential immunotherapies to manipulate ILC responses will be briefly discussed.

## 2 Response of ILC Subtypes to Bacterial Infections

**Figure 1:**
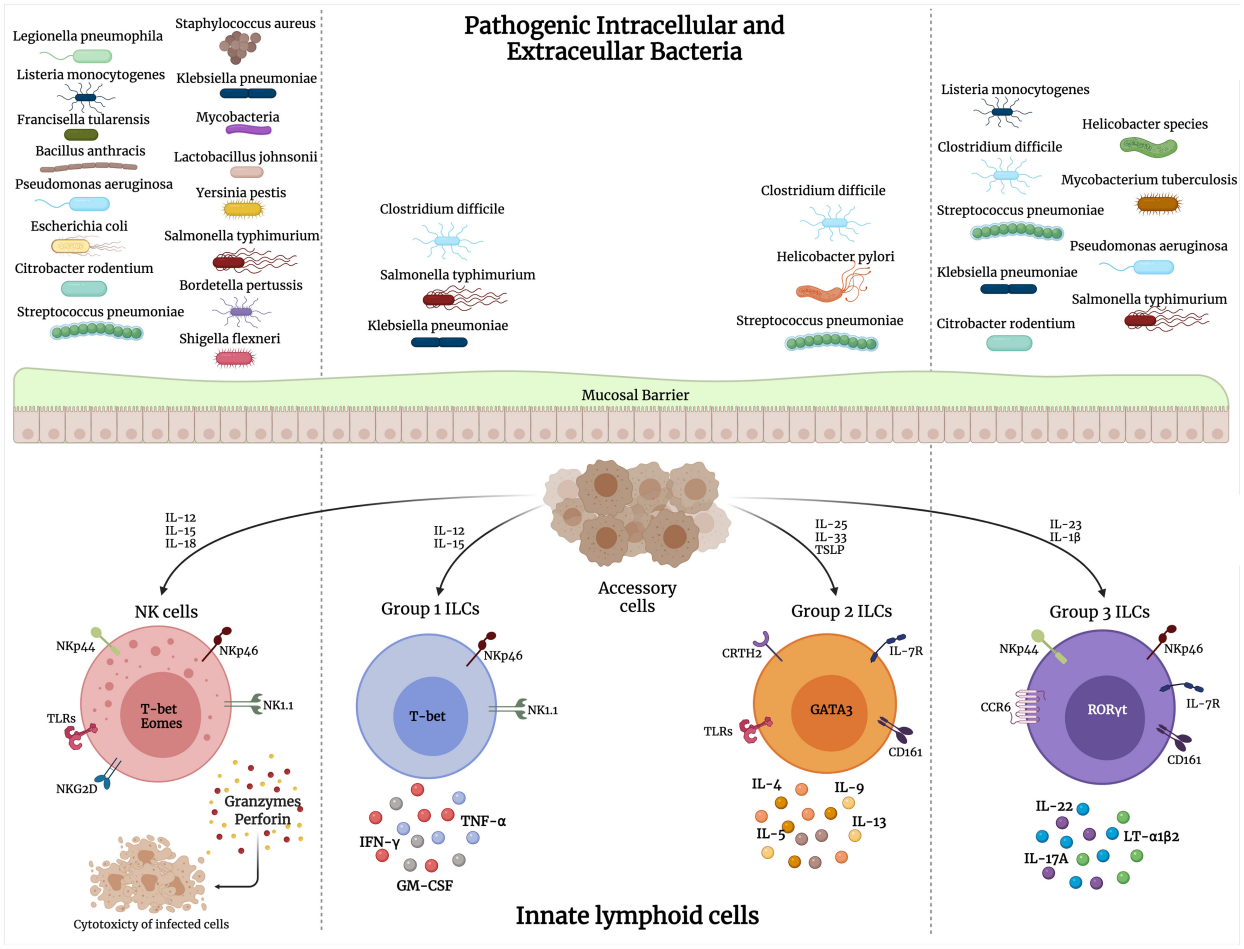
Interaction of pathogenic intracellular and extracellular bacteria with different groups of innate lymphoid cells (ILCs). Upon bacterial stimulation, various accessory cells secrete cytokines that activate ILCs to perform their direct cytolytic activity on bacteria/infected cells, or release cytokines that mediate bacterial clearance.

ILCs are a crucial component of the innate and adaptive immune responses to bacterial infections. The type of pathogen largely determines the selective ILC response during infections. As pointed out earlier, intracellular bacteria elicit mainly an ILC1 and some ILC3 response, while extracellular bacterial and fungal infections stimulate primarily the ILC3 subset. ILC2s are involved in response to parasitic infections and tissue repair in response to viral-induced tissue injury. ILC secretion of a variety of chemokines and cytokines results in the recruitment of other immune players and amplification of the inflammatory response against these pathogens. There has been extensive research on the role of ILCs in viral and parasitic infections as reviewed in (Diefenbach, 2013; Vivier et al., 2018; Hildreth and O'Sullivan, 2019; Hirose et al., 2019; Loser et al., 2019; Panda and Colonna, 2019; Seo et al., 2020). Here, we discuss the involvement of the ILC subsets across various bacterial infections (**Figure 1**).

### 2.1 Group 1 ILCs

#### 2.1.1 NK Cells

Numerous studies have reported that intracellular and extracellular bacteria activate NK cells (Harrington et al., 2007; Small et al., 2008; Souza-Fonseca-Guimaraes et al., 2012; Schmidt et al., 2016). These include *Listeria monocytogenes, Francisella tularensis, Chlamydia pneumoniae*, and* Yersinia enterocolitica* as well as sepsis (Bohn and Autenrieth, 1996; López et al., 2004; Berg et al., 2005; Thäle and Kiderlen, 2005; Etogo et al., 2008). The anti-bacterial potential of NK cells was reported in various bacterial infections, where NK cells were able to lyse *Shigella flexneri*, *Legionella pneumophila*, *Mycobacterium lepraemurium* or *Mycobacterium avium* infected monocytes (Klimpel et al., 1986; Blanchard et al., 1987; Katz et al., 1990; Denis, 1991). Also, NK cells have displayed bactericidal effects against macrophages infected with intracellular bacilli (Bermudez et al., 1990). Activated NK cells were found in the airways of mice infected with *Staphylococcus aureus* or *Bordetella pertussis* where they were found to play crucial roles in bacterial clearance (Byrne et al., 2004; Small et al., 2008). The immunoregulatory role of NK cells include cytokine production, including IFN-γ, GM-CSF, and TNF-α, that contribute to the inflammatory environment during an infection (Huntington et al., 2007; Lünemann et al., 2009). Further, upon activation with cytokines such as IL-12, IL-15 or IL-18, NK cells can also release IL-6, IL-10, transforming growth factor (TGF)-β, IL-17 and IL-22 (Jewett et al., 1996; Cella et al., 2009; Perona-Wright et al., 2009; Hall et al., 2010; Passos et al., 2010), as well as various chemokines (Maghazachi and Al-Aoukaty, 1998; Fehniger et al., 1999; Maghazachi, 2010). Additionally, the cytotoxic molecules released by NK cells (perforin and granzymes) possess an anti-bacterial effect on intracellular and extracellular bacteria such as *L. monocytogenes*, *Salmonella typhimurium, Bacillus anthracis, Escherichia coli, S. aureus* and *Mycobacterium tuberculosis* (Stenger et al., 1998; Ernst et al., 2000; Endsley et al., 2009; Gonzales et al., 2012; Lu et al., 2014). Another cytotoxic molecule secreted by NK cells is granulysin that disrupts the membrane of bacteria and has potent anti-microbial activity against various gram-positive and gram-negative bacterial species (Krensky and Clayberger, 2009; McSharry and Gardiner, 2010). A possible mechanism of action of the bactericidal granulysin could be via inducing lesions and distortions in the bacterial membrane (Stenger et al., 1998). Further, other studies reported that granulysin interferes with oxidative metabolism and energy generation by the bacteria (Krensky and Clayberger, 2009). For instance, granulysin was reported to directly kill the extracellular *M. tuberculosis* by altering the membrane integrity of the bacillus, while it further decreased the viability of intracellular *M. tuberculosis* when combined with perforin (Stenger et al., 1998).

During an infection, the crosstalk between NK cells and other accessory cells, such as DCs or macrophages, enables them to perform their anti-microbial activity. There was a reported indirect activation of NK cells by various types of bacteria including *L. monocytogenes, S. aureus, Lactobacillus johnsonii, *and *Mycobacterium* infections. This could be due to the recognition by mature DCs and secretion of cytokines such as IL-12, IL-18, and type-1 interferons (Nomura et al., 2002; Newman et al., 2006). IL-18 is a pro-inflammatory cytokine that is crucial in restriction of bacterial growth as reported by studies with hindrance of neutrophil-mediated lung damage in *M. tuberculosis* infected mice as well as *Legionella pneumophila* infection (Spörri et al., 2008; Schneider et al., 2010). Moreover, IL-18 triggered NK cell activity and IFN-γ production upon administration of lipopolysaccharide (LPS) and in *Propioni-bacterium acnes* infection (Takeda et al., 1998). Also, IL-18 was found to trigger γδT cells to produce IL-17A, which promotes IFN-γ production by NK cells upon injection of LPS (Andrews et al., 2011).

The secreted mediators by NK cells possess potent anti-bacterial activity against a variety of gram-negative and gram-positive bacteria (Garcia-Peñarrubia et al., 1989). At the same time, NK cells are able to halt their own activation in fighting bacterial infections including *L. monocytogenes *and* Yersinia pestis*. For instance, NK cells can secrete the regulatory cytokine IL-10 which inhibits IL-12 secretion by DCs (Perona-Wright et al., 2009). Such a reaction could be required to prevent immune pathology during systemic infections. In enteric bacterial infections, it was found that NK cells play a vital role in bacterial clearance such as that observed in *Citrobacter rodentium* infection (Hall et al., 2013). This could be done through cytokine release, direct cytotoxic effects to *C. rodentium* and activation of other innate and adaptive immune cells, leading to prevention of bacterial dissemination into the systemic circulation (Hall et al., 2013). Similarly, this NK cell behavior was observed in the infection of the lungs with *M. tuberculosis* (Feng et al., 2006). Further, NK cells are able to control murine *M. tuberculosis *infections upon their activation with various cytokines, such as IL-12, IL-18, and IL-2 from CD4^+^ T cells (Evans et al., 2011). Another possible way of NK cell elimination of *M. tuberculosis* is through the secretion of IL-22 upon stimulation with IL-15 and IL-23 (Dhiman et al., 2009). Upon activation with IL-12, NK cells secrete IFN-γ, leading to the elimination of bacterial infections including those caused by *Y. enterocolitica, S. typhimurium*, and *L. monocytogenes*, as well as stimulation with LPS (Hunter et al., 1995; Bohn and Autenrieth, 1996; Mastroeni et al., 1996; van de Wetering et al., 2009). Furthermore, macrophages promote NK cell activation by upregulation of surface CD69 upon exposure to LPS as well as NK cell production of IFN-γ in *Legionella pneumophila* and *L. monocytogenes* infections (Blanchard et al., 1988; Wherry et al., 1991; Scott et al., 2004). In turn, IFN-γ can favor the production of IL-12 from macrophages as reported in *Mycobacterium bovis* infection (Matsumoto et al., 1997). Being the main cytokine released by NK cells, IFN-γ, was reported to play a crucial role in fighting bacterial infections by inducing macrophage-mediated phagocytosis of bacteria or infected cells (McSharry and Gardiner, 2010; Horowitz et al., 2012). This was highlighted by a study where IFN-γ deficient mice exhibited an increase in the bacterial load and impairment in the anti-bacterial immune response upon infection with *Legionella pnemophilia, L. monocytogenes*, or mycobacteria (Cooper et al., 1993; Huang et al., 1993; Spörri et al., 2006). Moreover, IFN-γ could affect chemokine production and recruitment of immune cells to *S. aureus* infected tissues (McLoughlin et al., 2008). Besides, IFN-γ affected the availability of iron that is an essential nutrient needed for bacterial replication, as reported in *S. typhimurium* infected macrophages (Nairz et al., 2008). In addition, NK cells and specifically IFN-γ can modulate the maturation and activation of other adaptive immune cells populations by regulating the antigen presentation function (Degli-Esposti and Smyth, 2005; Nedvetzki et al., 2007; Hall et al., 2010; Horowitz et al., 2012). Another possible mechanism of host protection by IFN-γ, is the formation of granuloma post-infection with intracellular bacteria, such as *Mycobacterium avium* and *Francisella tularensis*, thus isolating infectious lesions (Smith et al., 1997; Bokhari et al., 2008).

Another way of activation of NK cells is through their expression of pathogen recognition receptors (PRRs) which bind to pathogen-associated molecular patterns (PAMPs) on the surface of certain bacteria (Chalifour et al., 2004; Souza-Fonseca-Guimaraes et al., 2012; Adib-Conquy et al., 2014). In response, several cytokines are released by NK cells that contribute to the cytokine storm present in infections (Cavaillon et al., 2003; Hargreaves and Medzhitov, 2005). Also, this could lead to production of α-defensins which are anti-microbial peptides that cause bacterial death by disruption of the bacterial membrane (Agerberth et al., 2000; Doss et al., 2010). This may also represent a direct cytotoxic pathway involved in NK cell-mediated protection against bacteria such as that observed in *C. rodentium*. Furthermore, NK cells can secrete cathelicidin (LL37) as well as indoleamine 2,3-dioxygenase (IDO) and nitric oxide (NO), that possess anti-bacterial effects and can limit infections (Agerberth et al., 2000; Bogdan, 2001; Zelante et al., 2009).

Among these PRRs are toll-like receptors (TLRs), a family composed of ten receptors that recognize various microbial components (Akira and Sato, 2003). TLR expression was controversial in literature depending on the investigated NK cell population (Adib-Conquy et al., 2014). However, the majority of the TLR family (TLR1-9) are expressed on NK cells (Hornung et al., 2002; Lauzon et al., 2006; Muhammad et al., 2019). It is noteworthy that post-translational modifications in NK cells could be the reason for the variability in TLR expression in literature. TLR2 on NK cells was found to be stimulated by peptidoglycan from *M. tuberculosis* and protein A from *Klebsiella pneumoniae* (KpOmpA) (Chalifour et al., 2004; Esin et al., 2013). On the other hand, the anti-bacterial role of NK cells could be triggered upon binding of TLR4 with the FimH protein of *E. coli*, TLR5 binding to flagellin of *E. coli*, and TLR9 binding to bacterial unmethylated CpG motifs (Sivori et al., 2004; Tsujimoto et al., 2005; Mian et al., 2010; Esin et al., 2013). LPS, a major component of the outer membranes of gram-negative bacteria, was found to stimulate TLRs on NK cells, resulting in their activation (Schmidt et al., 2016). Further, TLR4 agonists stimulate the production of IFN-α/β, which contribute to the NK cell activation in bacterial infections (Nguyen et al., 2002). Furthermore, such type 1 interferon secretion is associated with the production of the chemokine CXCL10 from infected cells, which promotes the chemotaxis of NK cells (Lande et al., 2003). Previous work by Muhammad J.S. *et al.* reported that NK cells are stimulated by LPS leading to the release of the proinflammatory cytokine IL-1β through pyroptosis signaling pathway (Muhammad et al., 2019). As mentioned earlier, another possible way of NK cell mediated bacterial elimination is through their indirect interaction with DCs. For instance, a study by Oth T. *et al.* demonstrated that beside the direct sensing of bacterial pathogens by NK cells and the induction of their cytotoxic capacity, there is also an enhancement of NK cell-mediated help for DC maturation (Oth et al., 2018). This was mainly attributed to the soluble factors released by PAMP-triggered NK cells, especially IFN-γ, that was able to intensify the pro-inflammatory cytokine response of DCs.

Other PRRs include the nucleotide-binding and oligomerization domain (NOD)-like receptors (NLRs) and the retinoic acid inducible gene I (RIG-I)-like receptors, that are expressed on NK cells. For instance, NOD1 and NOD2 receptors bind to motifs derived from peptidoglycan of gram-negative bacteria and gram-positive bacteria, thus promoting NK cell activation as indicated by CD69 expression and IFN-γ production. Also, NLRP3, the key element of an inflammasome, was reported to be expressed in NK cells (Qiu et al., 2011). Further, NLRP3 activation in macrophages during *Bordetella pertussis* infection, resulted in the production of IL-18 and IL-1β, thus promoting NK cell activation and proinflammatory response against the bacteria (Kroes et al., 2019).

NK cell receptors were reported to recognize and directly interact with host cell proteins as well as viral and bacterial proteins. For instance, studies have shown that the natural cytotoxicity receptor, NKp44, could directly bind to ligands on the surface of *M. tuberculosis* and *Pseudomonas aeruginosa* (Esin et al., 2008). Similarly, NK cells were able to recognize and directly interact with bacteria such as *Mycobacterium bovis BCG*, leading to their activation and release of cytokines such as IFN-γ and TNF-α, as well as cytolytic activity of target cells (Marcenaro et al., 2008). This was attributed to the expression and function of TLR2 on NK cells (Marcenaro et al., 2008). Besides, NKp46 was found to be a potential receptor for vimentin protein that is expressed on the surface of infected monocytes with *M. tuberculosis* (Vankayalapati et al., 2002; Garg et al., 2006). Additionally, UL16-binding proteins (ULBPs) and MHC class I polypeptide–related sequence A/B(MICA/MICB), ligands of NKG2D receptor were found to be expressed on infected monocytes, leading to NK cell activation to perform their cytolytic activity. Also, LPS-stimulated macrophages induce NK cell proliferation, IFN-γ production, and cytotoxicity as well as increase the surface expression of ULBPs 1, 2, 3 and MICA/B (Nedvetzki et al., 2007). Further, ULBP1 was upregulated in *M. tuberculosis*-infected monocytes and alveolar macrophages (Vankayalapati et al., 2005), while MICA was elevated on the surface of epithelial cells in *E. coli* infection (Tieng et al., 2002).

Bacteria release toxins that are so called superantigens, that activate NK cells. For example, the streptococcal pyrogenic exotoxin A (SPEA) was found to induce IFN-γ production as well as NK cell cytotoxic activity (Cavaillon et al., 1982; Sacks et al., 1991; Dobashi et al., 1999). Similarly, the staphylococcal enterotoxin B and the exotoxin A produced by *Pseudomonas aeruginosa* were reported to activate NK cells and their function including cytotoxicity and IFN-γ release (D'Orazio et al., 1995; Mühlen et al., 2004).

NK cells could be a friend or foe for bacterial infections, depending on the environment. In fact, the detrimental effects of NK cells in fighting bacterial infection were previously reported. Some studies claim that depleting NK cells may be beneficial and result in bacterial clearance including *E.coli, Streptococcus pneumoniae* and *Pseudomonas aeruginosa* (McSharry and Gardiner, 2010). Also, excessive LPS stimulation of IFN-γ production by NK cells could lead to an uncontrolled secretion of pro-inflammatory cytokines that could lead to lethal septic shock (Doherty et al., 1992; Emoto et al., 2002; Sherwood et al., 2004; Etogo et al., 2008; Souza-Fonseca-Guimaraes et al., 2012; Adib-Conquy et al., 2014). Therefore, a balance should be maintained in the immune response in order to have a beneficial anti-bacterial effect. On the contrary, NK cell cytotoxic activity was found to be impaired in patients with sepsis (Maturana et al., 1991). Additionally, some pathological effects of IFN-γ were reported in bacterial infections. For example, it was found that IFN-γ could cause death in polymicrobial peritonitis, *P. aeruginosa* infection and upon administration of LPS in mice (Miles et al., 1994; Heremans et al., 2000; Murphey et al., 2004).

#### 2.1.2 Other Group 1 ILCs

Besides NK cells, group 1 ILCs have shown to be central players in the protection against bacterial infections (Beck et al., 2020). ILC1s are potent producers of TNF-α and IFN-γ upon stimulation with IL-12, IL-15 and IL-18, that allow them to play key roles in immune protection and chronic inflammation (Fuchs, 2016). Additionally, such cytokine stimulation, and especially IL-15 aids in their development and contribution to enhanced immunity against infectious diseases including several viruses and bacteria (Diefenbach et al., 2014; Klose et al., 2014; Kwon et al., 2019; Poniewierska-Baran et al., 2019). For example, *Rag1*−/− mice lacking T and B cells when infected with *Clostridium difficile* bacteria, displayed ILC1-associated proteins such as IFN-γ, TNF-α and nitric oxide synthase (NOS)2. On the contrary, mice lacking all innate lymphoid cells, especially ILC1s, witnessed an increased susceptibility to *C. difficile* infection (Abt et al., 2015). *In vitro* studies of the gram-negative and pathogenic *S. typhimurium* infection revealed that infection of human colonic lamina propria cells led to IFN-γ production by ILC1s and NK cells (Klose et al., 2013). Similarly, the ILC response and their respective cytokines such as TNF, IL-23, IL-17, and IFN-γ were critical players in the clearance of *Klebsiella pneumoniae* infection (Moore et al., 2002; Xiong et al., 2016). Also, there was a significant decrease in ILC1 and ILC3 populations in the peripheral blood of sepsis patients (Cruz-Zárate et al., 2018). On the contrary, another study reported an increase in ILC1 but a decrease in ILC3 in the peripheral blood of patients with septic shock (Carvelli et al., 2019). Such a discrepancy in the studies could be attributed to the plasticity of ILC populations.

### 2.2 Group 2 ILCs

Analogous to Th2 cells, ILC2s mediate a type 2 immune response. They produce characteristic Th2 cytokines, including IL-4, IL-5, IL-9 and IL-13 (Moro et al., 2010; Neill et al., 2010; Price et al., 2010). Human ILC2s are characterized by the expression of chemoattractant receptor, CRTH2 and NK cell marker, CD161 (Mjösberg et al., 2011) and respond to cytokine cues including IL-25, thymic stromal lymphopoietin (TSLP), and IL-33. ILC2s are known to play a vital role in extracellular parasitic infections (Bouchery et al., 2015), allergic diseases such as asthma, rhinitis, atopic dermatitis (Tojima et al., 2019; Akdis et al., 2020), and tissue repair (Monticelli et al., 2015; Rak et al., 2016).

Recently, their role in bacterial infection is gaining interest. ILC2 activity has been reported to be regulated by various triggers, including signaling via cytokine receptors, lipid-, metabolite-driven, neuro-immune and microRNA modulation (Burrows et al., 2019). IL-33 production by the gut and lung epithelium is important for maintaining gut and lung homeostasis through the recruitment of ILC2s. Tuft cells are important responders to bacterial presence in the intestinal mucosa. During type 2 immune responses, tuft cells are capable of influencing the gut microbiome by regulating the intestinal ILC2-epithelial response circuit. Upon chemosensory-like sensing of pathogens, tuft cell-derived IL-25 triggers IL-13 secretion by resident ILC2s which in turn activated goblet cells to release mucus that aided in the clearance of bacterial pathogens (von Moltke et al., 2016). Metabolite-triggered small intestinal tuft cell-ILC2 circuit also orchestrated epithelial remodeling in the small intestine thereby shaping epithelial responses to intestinal pathogen that impaired their infestation (Schneider et al., 2018). The surface expression of TLR1, 4 and 6, on ILC2s enables them to respond to TLR ligands by secreting cytokines, such as IL-5 and IL-13, and inducing the production of immunoglobulins IgM, IgG, IgA, and IgE by B cells, which are important in shaping the microbial flora (Maggi et al., 2017).

Gastric *Helicobacter pylori* infection of the gut is a highly prevalent condition. *H. pylori* infection induces a skewed type 2 immunity and immunosuppressive microenvironment that is mediated by ILC2s (Li et al., 2017a). Commensal stomach bacteria favor an ILC2 environment by inducing the production of IL-7 and IL-33 cytokines, thereby making it the predominant ILC subset in the stomach (Satoh-Takayama et al., 2020). ILC2-dependent IgA response protected the stomach by eliminating IgA-coated bacteria including pathogenic *H. pylori* (Satoh-Takayama et al., 2020). The increasing prevalence of antibiotic heteroresistance among *H. pylori* strains is a matter of grave concern (Rizvanov et al., 2019). Better understanding of the underlying involvement of ILC2s may therefore, lead to better therapeutic approaches.

Similarly, *Clostridium difficile* colonizes the epithelial cells of the gut, releasing toxins that triggers cell death pathways and colonic inflammation. *C. difficile* infection upregulated IL-33 production that in turn activated ILC2s leading to prevention of epithelial death and disruption (Frisbee et al., 2019). Therefore, IL-33-mediated ILC2 activation is a key defense mechanism against *C. difficile* colitis.

Lungs constitute a unique organ as they are under constant exposure to the external environment since birth. The immunological milieu in the lungs is specialized to protect them from damage and infection. In a study by Saluzzo S. *et al*., the epithelium-derived IL-33 was found to be increased after the first day of life in newborn mice and this was closely followed by IL-13 secretion from ILC2s (Saluzzo et al., 2017). The homeostatic role of ILC2s in the lungs entailed the recruitment of alveolar macrophages and their IL-13 driven-polarization to an anti-inflammatory M2 phenotype (Saluzzo et al., 2017). However, this led to a delayed immune response to *S. pneumoniae* infection in mice during adult life.

### 2.3 Group 3 ILCs

Abundantly found in the intestines, ILC3s are indispensable in the maintenance of intestinal immunity and microbiota-host homeostasis. Microbial stimulation leads to the development of these cells after birth and various environmental signals, such as bacterial and dietary metabolites, regulate ILC3 differentiation and function (Qiu et al., 2012; Mielke et al., 2013). ILC3s are characterized by their production of IL-22 and/or IL-17, and are thus, the innate equivalent of Th17 cells. IL-22, however, is the predominant cytokine produced by these cells which contributes to intestinal homeostasis.

ILC3s are crucial in wading off bacterial infections as well as in the interactions with commensal bacteria. Occasionally, commensal bacteria can penetrate the mucosal barrier and thus, the human body has evolved mechanisms to re-establish homeostasis by minimizing inflammation. The intestinal microbiome interacts indirectly with ILC3s by promoting crosstalk between innate myeloid and lymphoid cells (Mortha et al., 2014; Gury-BenAri et al., 2016; Castleman et al., 2019). Both commensal as well as pathogenic bacteria induce accessory cells in the milieu, such as CD11c^+^ myeloid DCs to generate IL-23 and IL-1β which then contribute to IL-22 secretion from ILC3s (Castleman et al., 2019). In turn, IL-22 helps restrict the dissemination of commensal bacteria by inducing the expression of anti-microbial peptides (AMPs), including peptides of the S100 family, RegIIIβ and RegIIIγ (Sonnenberg et al., 2011a). AMPs possess potent anti-microbial as well as anti-biofilm activity. Also termed host defense peptides, these positively charged amphipathic molecules selectively target a broad spectrum of bacteria and kill them via several mechanisms. Their main mechanism of action is attributed to disrupting the bacterial cell membrane causing cell lysis and death. Additionally, they also form transmembrane channels in the membrane initiating cytoplasm leakage and cell death. Furthermore, they have demonstrated intracellular inhibitory activities by inhibiting essential intracellular functions by binding to intracellular proteins or nucleic acids (Le et al., 2017). In addition to their involvement in the innate immune response to extracellular bacteria, these IL-22 producing cells promote selective anatomical containment of lymphoid-resident commensal bacteria thereby preventing systemic inflammation (Cella et al., 2009; Sonnenberg et al., 2012; Hepworth et al., 2013). Along with IL-22 production, they also secrete IL-17 and regulate adaptive Th17 responses (Hepworth et al., 2013). GM-CSF is another cytokine that is produced by both mice and human ILC3s (Mortha et al., 2014). While the cell surface expression of NKp46 characterizes ILC3s in mice, NKp44 expression is observed in humans (Cupedo et al., 2009; Sanos et al., 2009). ILC3s also express CD127 (IL-7 receptor α-chain) and CD161. Although ILC3s are predominantly dependent on the transcription factor RORγt, T-bet expression is observed in a subset of NKp46^+^ cells and is essential for IL-22 and IFN-γ production in these cells (Sciumé et al., 2012; Klose et al., 2013; Rankin et al., 2013). Further, expression of major histocompatibility complex class II (MHC class II) by CCR6-expressing lymphoid tissue inducer (LTi)-like ILC3 is another mechanism by which they downregulate pathological CD4^+^ T cell responses against commensal bacteria thereby limiting spontaneous intestinal inflammation (Hepworth et al., 2013).

Infection by *S. typhimurium* causes diarrhea and gastroenteritis. IFN-γ response of innate origin is crucial in restricting the growth of *S. typhimurium* (Muotiala and Mäkelä, 1990; Songhet et al., 2011). In addition to expanding the armory in the fight against intracellular pathogens (Yrlid et al., 2001), IFN-γ also modulates goblet cell function during *S. typhimurium* infection (Songhet et al., 2011). Mucosal RORγt^+^ ILCs are emerging as important players in the immunity against intestinal infections. Studies indicate that ILC3s and not NK cells are the major source of IFN-γ during *S. typhimurium* infection (Vonarbourg et al., 2010; Klose et al., 2013). Graded expression of T-bet was found to determine the fate of a distinct lineage of CCR6- RORγt^+^ ILCs by influencing their expression of IFN-γ and the natural cytotoxicity receptor NKp46 (Klose et al., 2013). During *Salmonella enterica* infection, IFN-γ from these CCR6^-^ RORγt^+^ ILCs was essential for the secretion of mucus-forming glycoproteins that ensures epithelial barrier integrity (Klose et al., 2013). IL-23 orchestrated inflammatory mucosal response during *S. typhimurium* infection. This involved the early production of IL-22 and IL-17A by T cells and ILC3s (Godinez et al., 2009; Siegemund et al., 2009).

Another possible anti-bacterial mechanism of ILC3s is through the induction of the expression and secretion of lipocalin-2 via IFN-γ and IL-22 at barrier surfaces (Sonnenberg et al., 2010; Zhao et al., 2014). This limits bacterial growth by sequestrating the iron scavenged by bacteria during infection (Flo et al., 2004). Commensal bacteria are known to induce fucosylation of intestinal epithelial cells by adding L-fucose to glycolipids and glycoproteins on epithelial cells. The fucose moiety serves as a dietary carbohydrate for these bacteria, where they are metabolized into beneficial metabolites such as short-chain fatty acids. In addition, ILC3s induce the IL-22-mediated intestinal expression of fucosyltransferase 2 and subsequent epithelial fucosylation that promote barrier integrity in the intestinal tract (Goto et al., 2014). ILC3-mediated intestinal epithelial cell glycosylation reduces the susceptibility and improves host tolerance to *S. typhimurium* infection.

NK-derived IFN-γ is largely implicated in controlling the dissemination of intracellular bacteria such as *L. monocytogenes.* Oral infection by *L. monocytogenes* was observed to induce IFN-γ production by NKp46^+^ RORγt^-^ ILCs or NK cells and IL-22 production by NKp46^+^ RORγt^+^ ILC3s as well (Reynders et al., 2011).

*Citrobacter rodentium* is known to cause acute infection of the colonic epithelium leading to mild colitis. Infection with *C. rodentium* is associated with IL-23 dependent CD4^+^ LTi cell responses (Sonnenberg et al., 2011b). Further, these cells are early responders to infection through the production of IL-22. Depletion of CD4^+^ LTi cells led to a decline in the expression of infection-induced IL-22 and anti-microbial peptides that impaired innate immunity in the intestine. While ILC3s are important responders in the initial phase of *C. rodentium* infection, B lymphocytes and CD4^+^ T cells are crucial for resolution of *C. rodentium* infection (Simmons et al., 2003). In addition to activation of the surface receptors, various other mechanisms such as surrounding phagocytes, diet- and bacteria-derived metabolites can contribute to ILC3 activation during *C. rodentium* infection (Beck et al., 2020). LTi-ILC3s are equipped with a wide variety of receptors, including MHCII, NK cell receptor P1 (NKR-P1R), G-protein-coupled receptors (GPCRs) (GPR183, free fatty acid receptor 2 (Ffar2)), aryl hydrocarbon receptor (AHR) that sense environmental cues to mount an appropriate response against *C. rodentium* when triggered by the pathogen (Lee et al., 2011; Chu et al., 2018; Li et al., 2018; Chun et al., 2019; Melo-Gonzalez et al., 2019). Myeloid-ILC3 crosstalk also shapes the ILC3 response against *C. rodentium* infection. For example, the release of CXCL16 from DCs activates CXCR6 signaling in ILC3s stimulating the release of IL-22 and secretion of antimicrobial peptides (Longman et al., 2014; Satoh-Takayama et al., 2014). Further, depletion of the chemokine receptor CX_3_CR1 led to reduced expression of IL-22, antimicrobial peptides RegIIIβ and RegIIIγ, and subsequently delayed clearance of *C. rodentium* (Manta et al., 2013). Similarly, depletion of these CX_3_CR1^+^ mononuclear phagocytes led to increased severity of colitis and mortality upon *C. rodentium* infection (Longman et al., 2014). In addition to nutrient sensing AHR, dietary vitamin A promotes intestinal homeostasis by recruiting immune cells and improving the integrity of the mucosal barrier (Li et al., 2017b; Xiao et al., 2019). Furthermore, the active vitamin A metabolite retinoic acid regulates the transcription factor RORγt thereby controlling the ILC3 response against *C. rodentium* infection (Goverse et al., 2016).

*C. difficile* is an opportunistic enteric pathogen that causes infection upon antibiotic-induced gut microbiota alterations. While ILC1s are critical for protection against acute *C. difficile* infection, ILC3s also play a supporting role where their depletion of IL-22 production was associated with a minor contribution to resistance (Abt et al., 2015). Also, a recent study demonstrated the role of short-chain fatty acids (SCFAs), in particular acetate, in ameliorating the infection by activating the free fatty acid receptor 2 (FFAR2) on ILC3s and neutrophils (Fachi et al., 2020). This ligand-receptor signaling led to increased neutrophil-mediated inflammasome activation and release of IL-1β, which boosted IL-1R expression on ILC3s and IL-22 production.

Non-gastric *Helicobacter* species such as *Helicobacter apodemus* and *Helicobacter typhlonius*, while activating ILCs and inducing gut inflammation, were found to negatively regulate RORγt^+^ ILC3s and weaken their proliferative capacity in immunocompromised mice (Bostick et al., 2019). Further, antigen-presenting ILC3s through their interaction with T follicular helper cells (Tfh) and B cells limited mucosal IgA responses to *H. typhlonius* in order to preserve mucosal-dwelling commensal microbiota (Melo-Gonzalez et al., 2019).

In response to *S. pneumoniae* infection, the release of IL-23 from DCs led to rapid accumulation of ILC3s in the lungs and their activation in an MyD88-dependent manner leading to IL-22 secretion (Kinnebrew et al., 2012; Van Maele et al., 2014). Further treatment with TLR5 agonist flagellin exacerbated ILC3-mediated IL-22 production, that helped provide defense against lethal infection (Van Maele et al., 2014). Furthermore, intestinal commensal bacteria protect neonatal mice against bacterial pneumonia immediately after birth by directing ILC3s influx into the lungs and IL-22-dependent host resistance to pneumonia (Gray et al., 2017). ILC3-derived IL-22 and IL-17 have also been implicated in host defense against *Klebsiella pneumoniae*, a bacteria that displays high-level of acquired antibiotic resistance (Chen et al., 2016; Murakami et al., 2016). During *K. pneumoniae* infection in mice, inflammatory monocytes rapidly migrate to the lung and secrete TNF, leading to the increased activation of IL-17-producing ILCs (Xiong et al., 2016). IL-17 and IL-22-producing ILC3s are essential for host response and defense against chronic pulmonary infection caused by *Pseudomonas aeruginosa*, possibly by moderating neutrophil-mediated lung damage (Bayes et al., 2016; Broquet et al., 2017). The crosstalk between myeloid cells and ILCs promotes clearance of pneumonia.

ILC3s are known to mediate an early protective response to tuberculosis, and in particular to *M. tuberculosis* (Ardain et al., 2019). Acute infection with pulmonary tuberculosis is associated with a depletion of circulating ILC subsets which are later restored upon treatment. In response to infection, ILC subsets, in particular ILC3s, accumulate in the lungs resulting in a robust innate immune response in containing the infection (Ardain et al., 2019).

## 3 Dysregulation of ILC Subsets in Bacterial Infections

**Figure 2:**
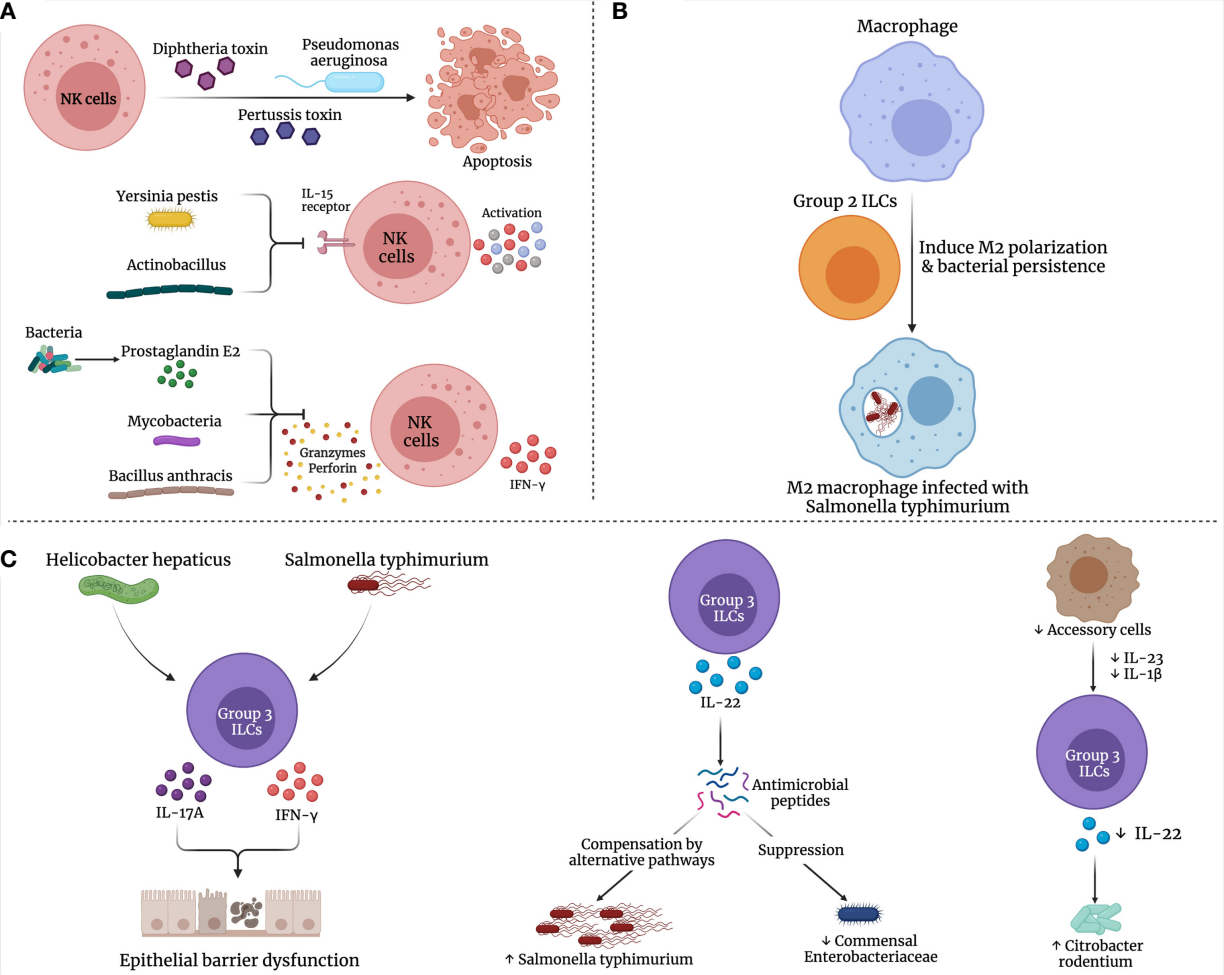
Possible mechanisms for dysregulation of ILCs in bacterial infections. Several bacterial species may manipulate the immune **(A)** NK cells, **(B)** Group 2, and **(C)** Group 3 ILCs, promoting bacterial persistence and inhibiting their elimination.

Bacterial pathogens have evolved strategies to evade, inhibit or manipulate the innate immune response to their advantage. Some of these strategies include subversion of antimicrobial peptides, modulation of innate immune signal transduction cascades, immune receptor localization and cytokine secretion (**Figure 2**). These mechanisms have been comprehensively reviewed in (Reddick and Alto, 2014).

### 3.1 NK Cells

Over the past decades, numerous studies have reported the strategies developed by bacteria to evade the NK cell response leading to bacterial persistence. For example, *Pseudomonas aeruginosa* could eliminate NK cells via phagocytosis-induced apoptosis (Chung et al., 2009). Further, toxins from various bacteria including diphtheria toxin, pertussis toxin and *P. aeruginosa* exotoxin A halt NK cell activity and promote NK cell apoptosis (Waters et al., 1990; Whalen et al., 1992; Michałkiewicz et al., 1999). *Bacillus anthracis* toxin was reported to inhibit NK cell cytotoxicity and IFN-γ secretion (Klezovich-Bénard et al., 2012). Similarly, cell wall components of mycobacteria affect DC maturation, hinder NK cell activity and IFN-γ production as well as promote immunosuppressive IL-10 secretion (Geijtenbeek et al., 2003). Also, leukotoxin produced by *Actinobacillus actino-mycetemcomitans* as well as the membrane virulence protein of *Yersinia pestis*, inhibit the expression of NK cell activation markers and IL-15 receptor, respectively (Shenker et al., 1994; Kerschen et al., 2004). Another approach by various bacterial species is promoting the production of prostaglandin E2 (PGE2), which suppresses NK cell response to cytokines, migration, IFN-γ production and cytolytic function, as illustrated in **Figure 2A** (Walker and Rotondo, 2004; Szymanski et al., 2012).

### 3.2 Other ILCs

Bacterial infections have evolved to cause substantial damage to epithelial barrier surfaces and its subsequent loss of protective function. Interestingly, this correlated with infection-induced perturbations in ILC frequency and function. The effect of ILCs on intestinal homeostasis is largely dependent on the bacteria-specific protective or deleterious cytokine response. In addition to fostering intestinal homeostasis in response to bacteria, ILCs may also promote an exaggerated inflammatory response. Inflamed mucosal tissues and inflammatory diseases of the gut [inflammatory bowel diseases (IBDs) and Crohn’s disease (CD)] were associated with increased frequency of IFN-γ/IL-17A-producing ILCs (Geremia et al., 2011; Bernink et al., 2013) and reduced frequency of IL-22-producing ILCs (Takayama et al., 2010; Bernink et al., 2015). The increased presence of IFN-γ at mucosal surfaces compromises the epithelial tight junctions and upregulates TNF-α receptor expression on epithelial cells, which induces intestinal epithelial barrier dysfunction (Wang et al., 2005; Beaurepaire et al., 2009).

*S. typhimurium* is known to manipulate macrophage polarization to a M2 state which enables their persistence within the macrophage leading to the establishment of persistent infection (Pham et al., 2020). ILC2s preferentially mediated the alternate activation of macrophages (Kim et al., 2019) that reportedly enhanced bacterial dissemination and long-term persistence in *S. typhimurium* infection (**Figure 2B**) (Eisele et al., 2013).

Depending on the cytokines in the milieu, ILC3s demonstrate plasticity in their effector cytokine production (Bernink et al., 2015). IFN-γ-producing ILC3s may contribute to the breakdown of the gut epithelial barrier. A compromised epithelial barrier promotes intrusion of gut bacteria into the lamina propria resulting in immune cell exposure to a wide variety of bacterial species at varying magnitudes and induction of potentially pathogenic immune responses (Pastorelli et al., 2013). The production of IFN-γ/IL-17 in response to *S. typhimurium* or *Helicobacter hepaticus* reportedly promotes bacteria-driven innate colitis (Buonocore et al., 2010; Klose et al., 2013). Therefore, IL-23-responsive ILC3s could also mediate intestinal immune-mediated pathology.

As discussed above, ILC3-derived IL-22 is induced in response to bacterial infections and plays an important role in host defense at mucosal surfaces. At the same time, IL-22 has been reported to suppress the commensal bacteria while promoting the colonization of the pathogenic bacteria (Behnsen et al., 2014). In the study by Behnsen, J. *et al.*, it was demonstrated that IL-22 induced AMPs, including lipocalin-2 and calprotectin, that are responsible for sequestering essential metal ions from microbes, were compensated in *S. typhimurium* by alternative pathways. Moreover, IL-22 preferentially boosted the colonization of *S. typhimurium*, while suppressing commensal *Enterobacteriaceae* species that are susceptible to AMPs (**Figure 2C**). Thus, the production of IL-22 by ILC3s is exploited by pathogenic bacteria to enhance their colonization on mucosal surfaces at the cost of their competing commensals.

Furthermore, the production of IL-23 and IL-1β by accessory cells such as mDCs, monocytes and macrophages, helps induce and regulate IL-22 secretion from ILC3s (Manta et al., 2013; Castleman et al., 2019). The depletion of CX_3_CR1^+^ phagocytes in mice reduced IL-22 expression in ILC3s, leading to increased microbial translocation and delayed clearance of *C. rodentium* (Manta et al., 2013). Thus, pathogenic bacteria may potentially exploit this mechanism by either reducing the frequency of accessory cells or depleting the cytokines responsible for IL-22 production by ILC3s in order to promote bacterial dissemination.

## 4 Possible Therapeutic Approaches Using ILCs in Bacterial Infections

Considering their relatively recent discovery, ILCs are now actively studied to decipher their contribution to immune response in health and disease, and to manipulate them for clinical benefit. However, therapeutic strategies targeting ILCs should follow after firmly establishing a unique and non-redundant protective or deteriorating role for ILCs in diseases. Variety of strategies are now available to therapeutically target ILCs, including cytokine administration, adoptive transfer, anti-cytokine antibodies, antibody depletion of ILCs, modulating ILC plasticity and/or function, inhibiting ILC migration and function, and immune checkpoint modulation, as elaborately described in (Cobb and Verneris, 2021). With increasing preclinical and clinical studies, it is evident that ILCs play a role in the initiation, regulation and resolution of bacterial infections suggesting a potential beneficial role in therapeutically targeting ILCs in bacterial infections.

NK cells were heavily investigated and utilized as a therapeutic modality in various diseases including infections and cancer. Several studies have suggested using certain bacterial strains to activate NK cells, thus boosting their cytotoxicity effects against cancer cells. For example, the live vaccine strain BCG was proposed as a successful immunotherapy for bladder cancer due to the induced inflammation and recruitment of NK cells (Koga et al., 1988). Another example is *L. monocytogenes* infection which was found to initiate the anti-tumor NK cell response specifically against hepatic metastasis, due to their tropism in the liver (Yoshimura et al., 2007).

On the other hand, the role of ILC1s in Crohn’s disease highlighted its potential in being a therapeutic target. In CD patients, the frequency of CD127^+^ ILC1s was found to increase at the cost of ILC3s in inflamed intestinal tissues (Bernink et al., 2015). Here, the plasticity of ILCs can be exploited to differentiate IFN-γ-producing CD127^+^ T-bet^+^ c-Kit^−^ NKp44^−^ ILC1s into IL-22-producing NKp44^+^ ILC3s in the presence of IL-1β and IL-23, thereby re-establishing homeostasis which may demonstrate a therapeutic effect in Crohn’s disease.

While ILC3s are essential for the maintenance of gut homeostasis (Vivier et al., 2018), their dysregulation may also contribute to intestinal inflammation. For instance, increased colonic secretion of IL-17 and IFN-γ by ILC3s was associated with bacteria-driven innate colitis (Buonocore et al., 2010). In cases of redundancy between ILCs and T cells, neutralizing the common effector cytokines from ILCs and T cells may prove beneficial. Although targeting both IL-17 and IFN-γ appeared promising in pre-clinical studies (Buonocore et al., 2010), neutralizing these cytokines or blockade of IL-17R failed to demonstrate clinical efficacy against Crohn’s disease (Hueber et al., 2012; Kaser, 2014). In this case, targeting the upstream cytokines, such as IL-12 and IL-23 simultaneously or IL-23 alone, that stimulate the ILCs to produce IL-17 and IFN-γ may serve as a more effective strategy in treating patients with Crohn’s disease (Sands et al., 2017; Rutgeerts et al., 2018).

In addition, ILC3-derived GM-CSF is an important element in ILC-driven colitis, where they are responsible for the recruitment and maintenance of intestinal inflammatory monocytes (Pearson et al., 2016). The IL-23/GM-CSF–mediated autocrine feedback loop may sustain the crosstalk between myeloid cells and ILC3s. Further, GM-CSF may mobilize the ILC3s from within lymphoid aggregate cryptopatches into adjacent intestinal mucosa, as seen following the induction of colitis. Neutralization of GM-CSF prevented the egress of ILC3 from cryptopatches and bore promising results in mouse models (Pearson et al., 2016). However, GM-CSF may not be a straightforward target in IBD as GM-CSF governs clear host protective functions in the intestine (Bernasconi et al., 2010; Hirata et al., 2010).

In fact, several biological therapies that target ILCs are currently approved for the treatment of CD. Patients with CD are generally characterized by high intestinal levels of ILC1s and low ILC3s. Ustekinumab, monoclonal antibody against IL-12/23 p40, normalized the ILC frequencies, thereby contributing to intestinal mucosal healing in these patients (Li et al., 2016). In addition to ustekinumab, the inhibition of TNF-α and α4β7 integrin inhibitor (vedolizumab) are approved for Crohn’s disease (Crohn’s and Colitis Foundation, 2021). NCR^+^ ILC3s constitute an important source of intestinal IL-22 and were found to be reduced in the intestinal mucosa of CD patients in favor of pro-inflammatory ILC1s. In a study of 54 CD patients, increased ILC1 levels and significantly lower NCR^+^ ILC3 levels were detected at baseline (Creyns et al., 2019). However, biological therapy with anti-TNF, ustekinumab or vedolizumab was found to restore the NCR^+^ ILC3 levels to homeostatic proportions in the intestine. Interestingly, the circulating NCR^+^ ILC3s increased only in the anti-TNF and ustekinumab treatment groups but not with vedolizumab therapy. Taking into consideration the critical role of α4β7 integrin in the development and migration of ILCs (Sonnenberg and Artis, 2015; Tufa et al., 2020), the effect of vedolizumab on the frequency of peripheral ILC3s may suggest the lack of ILC homing from the blood to the gut (Forkel et al., 2019) but rather a selective inhibition of ILC migration from cryptopatches into intestinal mucosa. The biological efficacy of these therapeutics could thus, be attributed at least partially to its impact on ILC differentiation, migration and/or function, and hence re-establishing homeostatic intestinal conditions.

Various other therapies that are currently in the pipeline may also target ILCs. Multiple agents targeting IL-23 are in late-phase clinical trials for patients with CD and/or ulcerative colitis (Moschen et al., 2019) (NCT03650413). JAK inhibitors, through their ability to modulate IL-12 and IL-23 cytokine signaling may affect ILC plasticity and are promising candidates in trials. A monoclonal antibody targeting NKG2D is also being tested in a phase II clinical trial (NCT02877134). Since the gut microbiome is known to reciprocally regulate ILCs (Gury-BenAri et al., 2016), a small-molecule FimH antagonist, Sibofimloc, that was created to impede bacterial adherence to the gut, thus decreasing intestinal permeability and reducing innate immune activation, is being investigated in a phase II study (https://www.enterome.com/). Sphingosine-1-phosphate receptor 1 (S1PR1) regulates ILC egress from secondary lymphoid organs (Eken et al., 2019) and S1PR1 agonists, such as fingolimod, are expected to modulate their migration and function (Bell et al., 2018). With parallel action to regulatory T cells, regulatory ILCs (ILCregs) are proposed to promote the resolution of intestinal inflammation by suppressing the activation of ILC1 and ILC3 through IL-10 secretion. These therapeutic approaches have been reviewed in (Moschen et al., 2019).

## 5 Future Perspectives and Concluding Remarks

Bacterial molecules can activate ILCs via a direct and indirect mechanisms, hence highlighting the crucial roles for these innate cells in bacterial infections (**Table 1**). The past 10 years or so have been instrumental in shaping our understanding of the functional diversity of ILCs. Nevertheless, there are still a lot of open-ended questions that need to be answered to fully comprehend the complex roles of these cells in health and disease. ILCs are known to regulate key signaling circuits to establish tissue homeostasis; however, upon further dissection, new circuits may be revealed. Furthermore, unraveling the crosstalk between ILCs and the various other innate, adaptive immune players and non-hematopoietic cells may open up further avenues of research. Similarities in the molecular profiles between ILCs and T cells limits the specific targeting of ILCs without simultaneously affecting lymphocytes. ILCs are important mediators in the innate immune response to bacterial infections, particularly by regulating tissue-specific immunity. The traditional treatment regimens of bacterial infections are usually antibiotics. Over the past decades, bacteria have managed to develop evasion strategies including antibiotic resistance. Hence, alternative therapeutic modalities are needed. Being the first innate defense members in the anti-bacterial response, ILCs represent a new promising target for anti-bacterial therapy. Further research needs to focus on the immunoregulatory pathways controlled by ILCs and ways of therapeutically harnessing them.

**Table 1:** Response of different ILC groups in various intracellular and extracellular bacterial infections.

ILC Group	Bacterial Infections	Role/Response
NK cells	Shigella flexneri Mycobacterium lepraemurium Mycobacterium avium Listeria monocytogenes Salmonella typhimurium Bacillus anthracis Escherichia coli Staphylococcus aureus Mycobacterium tuberculosis Lactobacillus johnsonii Propioni-bacterium acnes Yersinia pestis Citrobacter rodentium Yersinia enterocolitica Mycobacterium bovis Legionella pneumophila Francisella tularensis Bordetella pertussis Pseudomonas aeruginosa Streptococcus pneumoniae	Cytotoxicity/Lysis via perforin and granzymes Cytotoxicity/Lysis via granulysin IFN-γ and TNF-α production Activation by dendritic cell released cytokines including IL-12, IL-18, and type-1 interferons Secretion of IL-10 to inhibit dendritic cell Activation of adaptive immune cells Secrete antibacterial mediators: cathelicidin, IDO, nitric oxide and α-defensins
ILC1s	Clostridium difficile Salmonella typhimurium Klebsiella pneumoniae	Production of IFN-γ, TNF-α, IL-23, IL-17 and nitric oxide synthase 2
ILC2s	Helicobacter pylori Clostridium difficile Streptococcus pneumoniae	Production of IL-4, IL-5, IL-9 and IL-13 cytokines
ILC3s	Salmonella typhimurium Salmonella enterica Citrobacter rodentium Listeria monocytogenes Clostridium difficile Helicobacter apodemus Helicobacter typhlonius Streptococcus pneumoniae Klebsiella pneumoniae Pseudomonas aeruginosa Mycobacterium tuberculosis	Secretion of cytokines including IL-17, GM-CSF, IL-22 Production of anti-microbial peptides such as RegIIIβ and RegIIIγ, calprotectin and lipocalin-2

## Author Contributions

NE and RR wrote the original manuscript. JH, AM, RH, and QH revised the manuscript. All authors contributed to the article and approved the submitted version.

## Conflict of Interest

The authors declare that the research was conducted in the absence of any commercial or financial relationships that could be construed as a potential conflict of interest.

## Publisher’s Note

All claims expressed in this article are solely those of the authors and do not necessarily represent those of their affiliated organizations, or those of the publisher, the editors and the reviewers. Any product that may be evaluated in this article, or claim that may be made by its manufacturer, is not guaranteed or endorsed by the publisher.
